# A Protocol to Assess Insect Resistance to Heat Waves, Applied to Bumblebees (*Bombus* Latreille, 1802)

**DOI:** 10.1371/journal.pone.0118591

**Published:** 2015-03-04

**Authors:** Baptiste Martinet, Thomas Lecocq, Jérémy Smet, Pierre Rasmont

**Affiliations:** University of Mons, Research Institute of Biosciences, Laboratory of Zoology, Place du Parc 20, 7000, Mons, Belgium; New Mexico State University, UNITED STATES

## Abstract

Insect decline results from numerous interacting factors including climate change. One of the major phenomena related to climate change is the increase of the frequency of extreme events such as heat waves. Since heat waves are suspected to dramatically increase insect mortality, there is an urgent need to assess their potential impact. Here, we determined and compared the resistance to heat waves of insects under hyperthermic stress through their time before heat stupor (THS) when they are exposed to an extreme temperature (40°C). For this, we used a new experimental standardised device available in the field or in locations close to the field collecting sites. We applied this approach on different Arctic, Boreo-Alpine and Widespread bumblebee species in order to predict consequences of heat waves. Our results show a heat resistance gradient: the heat stress resistance of species with a centred arctic distribution is weaker than the heat resistance of the Boreo-Alpine species with a larger distribution which is itself lower than the heat stress resistance of the ubiquitous species.

## Introduction

The current worldwide biodiversity undergoes one of the greatest mass species extinction in earth’s history [1]. The biodiversity decline results from numerous interacting factors including destruction and fragmentation of habitat, urban development, invasive species, pest plants/animals, pesticide use, and climate change. Among these factors, the climate change has been pointed out as one of the major causes of extinction in several groups of organisms [2–4].

The climate change encompasses modifications in mean temperature and precipitation throughout the year as well as changes in variability among years. Indeed, the current climate change is related to an increase of frequency of extreme event such as heat waves [5,6]. These heat waves have been linked to physiological perturbation (e.g. levels of HSP proteins, fecundity) [7] leading to increased mortality among several species as birds [8,9], flies [10], flying-foxes [11], weevils [12], butterflies [13] or bees [14,15]. Nevertheless, it remains difficult to predict heat wave consequences on species because each taxon has its own specific thermotolerance [12]. This places a premium on accurate determination of thermotholerance of each target species.

Here, we determine and compare the heat stress resistance under hyperthermic stress of small insects with a new experimental standardised device available in the field or in laboratory close to the field collecting sites. We apply this approach on different bumblebee species in order to predict consequences of heat waves.

## Materials and Methods

### Estimation of Heat stress resistance

Classically, methods to measure heat resistance have been borrowed from Uvarov [16] and Hutchison [17]. For instance, these methods were used to calculate the critical temperature to kill insects and other pathogen agents in foodstuff [16]. Here, we used a new static method [18–20] with a portable experimental device. Insects were exposed here to constant conditions: 40°C with constant humidity. The heat stress resistance of specimens has been estimated through their Time before Heat Stupor (THS).

### Test case

Bumblebees (Hymenoptera, Apidae, genus *Bombus*) are robust and hairy heterothermic bees with the ability of strong endothermy [21] that enables them to recolonize areas depopulated by glaciation events [22,23] and to live in some of the highest-elevation and most northern ecosystems. Their species diversity hotspots (mountains, Arctic, Subarctic and Boreal regions) are also regions hardest hit by climate change [24–27].

We sampled 144 males belonging to five different species from Eastern Pyrenees and North of Scandinavia (S1 Dataset): two taxa with an arctic distribution: (*B*.*alpinus* [n = 16], *B*.*balteatus* [n = 22], both belonging to *Alpinobombus* subgenus, [28]); three Boreo-Alpine (mountainous) taxa (*B*.*(Pyrobombus) monticola scandinavicus* [n = 15], *B*.*monticola rondoui* [n = 30], *B*.*(Psithyrus) flavidus* [n = 31]) and one widespread and ubiquitous species (*B*. *(Bombus sensu stricto) lucorum* [n = 30]) (S1 Dataset). Permissions for collection of Swedish samples were obtained via the *Länsstyrelsen Norrbotten*. No specific permits were required for the other described studies as collection did not occur in privately-owned, protected locations or protected species. The bioassay of this study does not require ethic permit as it applies to insects.

We used only males as they display simple and constant behaviour and they normally do not take shelter in thermoregulated underground nests as the females could do [21].

### Experimental device and protocol

We collected specimens in the field (S1 Dataset) and placed them in a fridge (WAECO CDF-35, 31L with a 12 V vehicle power supply) to keep them at 8°C (standby temperature according to Heinrich (1975, 1979) [21,29] in dark with food (i.e. BioGluc available *ad libitum*, Biobest NV, Westerlo, Belgium) during one day. After 24 hours, insects were placed individually in breakthrough Petri dishes.

**Fig 1:**
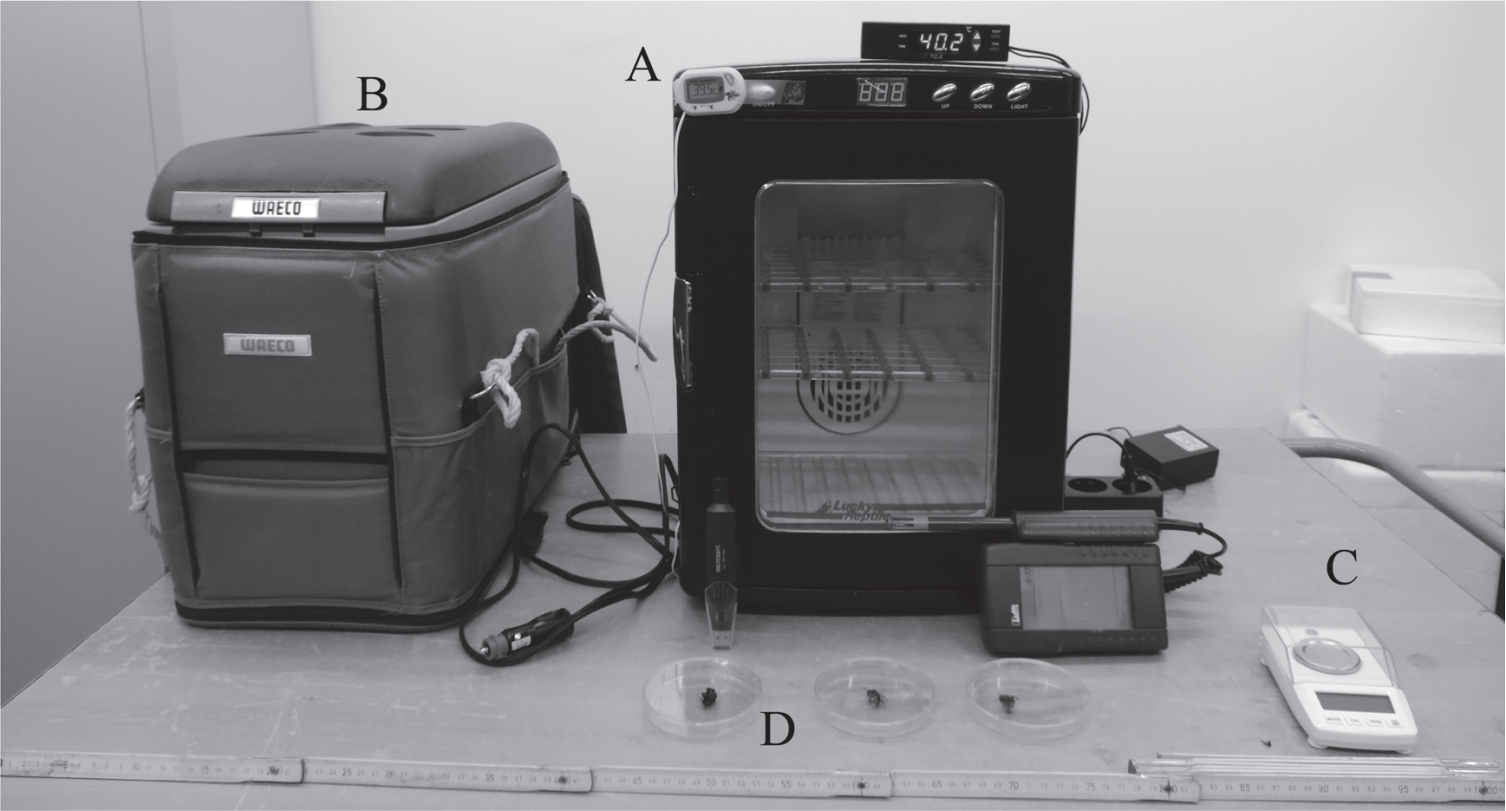
Picture of the experimental device. Picture of the experimental device: A) Incubator Herp Nursery II with its 12 V vehicle power supply, the datalogger (Voltcraft DL-181 THP USB Ambient Monitoring Data Logger), the psychrometer (Lufft C210), the digital thermometer (Zoo Med) and the thermostat (Lucky Reptil ThermoControl Pro II) B) Fridge WAECO CDF to keep bumblebees at 8°C with 12 V vehicle power supply, C) Scale Ace CT-50, D) Petri Dishes with tested bumblebees.

The Petri dishes with specimens were placed in the incubator (Herp Nursery II) at 40°C (Fig. 1). This incubator is a portable device where the temperature and humidity were controlled. This device can be used in the field when it is plugged into a 12 V vehicle power supply or other 12 V portable battery. The heating device includes a system of semiconductor thermoelectric Peltier elements (60 W) in an isolated oven of 25 dm^3^ (27cm x 23cm x 37cm) [30]. The incubator’s fan homogenises the temperature. The humidity of the enclosure is stabilised by free water disposed in the insert at the bottom of the incubator. A psychrometer (Lufft C210) and a datalogger (Voltcraft DL-181 THP USB Ambient Monitoring Data Logger) used to check the humidity. The resulting relative humidity of 50–60% corresponds to a normal rate in daytime (in summer) in the wild [31,32].

In order to improve the low accuracy of the air temperature regulation controller embedded in the Herp Nursery II, we added to the device a datalogger and a thermostat (Lucky Reptil ThermoControl Pro II, accuracy ± 0.1°C), including a thermometer, ables to stop and start the Peltier system of the incubator. We also included a digital thermometer (Zoo Med, accuracy ± 0.1°C) to check the stability of the air temperature inside the incubator. The temperature of 40°C was chosen in reference to the hottest temperature observed in 2011 in France [33]. Besides, even for the North of Fennoscandia this choice remains meaningful. Indeed, high air temperature can be measured even in Arctic, e.g. (an air temperature of 34°C has been recorded in Kevo in June 2013, [34]). With this experimental device the temperature remained constant [18]. In the wild, living male bumblebees make their courtship in sunlit open area [35] and they could actually experience harsher heat stress situations.

The individuals placed in the enclosure have been observed and monitored through the window in the door.

### Measure of Heat stress resistance

An insect is said to be entering into "dazed heat" or "heat stupor" [16,36] when it falls on its back, is unable to turn, and loses its normal reflexes [37]. The extremities are them shaken by muscle spasms [38] that appear just before death [16,18]. We distinguished the following sequence of activity: (i) normal activity (ii) important excitation, (iii) heat stupor, and (iv) death. The Time before Heat Stupor (THS) is measured for each individual tested with a chronometer (±1 minute). This was the time from insertion into the incubator to heat stupor.

Each Petri dish was flipped over at regular intervals (1–2 minutes) to check if the specimen was able to flip from up-down to normal position. The temperature loss caused by the opening of the door of the incubator is negligible and was estimated (with a datalogger) at less than 0.5°C. When the specimen became no more able to return in normal position, they have been assumed to be in “heat stupor”. The THS values have been recorded. Once in heat stupor; the insect was removed from the enclosure to recover. The percentage of mortality after experiment has been recorded. The dry weight of each species has been measured with a scale (Ace CT-50 Portable Miligram Scale, precision ± 0.001g) after lyophilisation (Lyovac GT2 LEYBOLD-HERAEUS in the laboratory of Zoology of the University of Mons (Belgium)

### Statistical analyses

From the results, Kruskal-Wallis analyses (Kruskal-Wallis test and Multiple comparison test after Kruskal-Wallis, “pgirmess” R-package, [39]) were performed to compare the different species altogether and boxplots were carried out using R (R Development Core Team, 2013) (“graphics” R-package, [40], “stats” R-package, [41]) to detect heat stress resistance differentiations between each species and between the two subspecies of *B*. *monticola* ssp. (*B*. *monticola rondoui* and *B*. *monticola scandinavicus*).

## Results

Our bioassays results in the death of 49.7% of individuals, while others recover feeding and flying again. Indeed the death percentage is about 50% in all species except *B*. *lucorum* (26%) (Table 1).

**Table 1:** Values of Time before heat stupor (THS), percentage of males bumblebees survivors (Survivors %) and median dry weight for five bumblebees species (*Bombus*).

**Species**	**Time beforeheat stupor (THS) (min)**			**Survivors (%)**	**Median dry weight (g)**
	**1^st^ Quartile**	**Mediane**	**3e Quartile**		
***Bombus lucorum* (n = 30)**	140	242	344	26	0.059 (n = 30)
***Bombus alpinus* (n = 16)**	20	31	40	56	0.074 (n = 11)
***Bombus balteatus* (n = 22)**	20	31	39	52	0.071 (n = 13)
***Bombus flavidus* (n = 31)**	71	82	90	48	0.060 (n = 31)
***Bombus monticola* sspp. (n = 45)**	70	91	132	58	0.033 (n = 28)
*B.m. scandinavicus*(n = 30)	70	94	133	61	0.041(n = 13)
*B.m. rondoui* (n = 15)	71	81	121	55	0.028 (n = 15)

**Fig 2:**
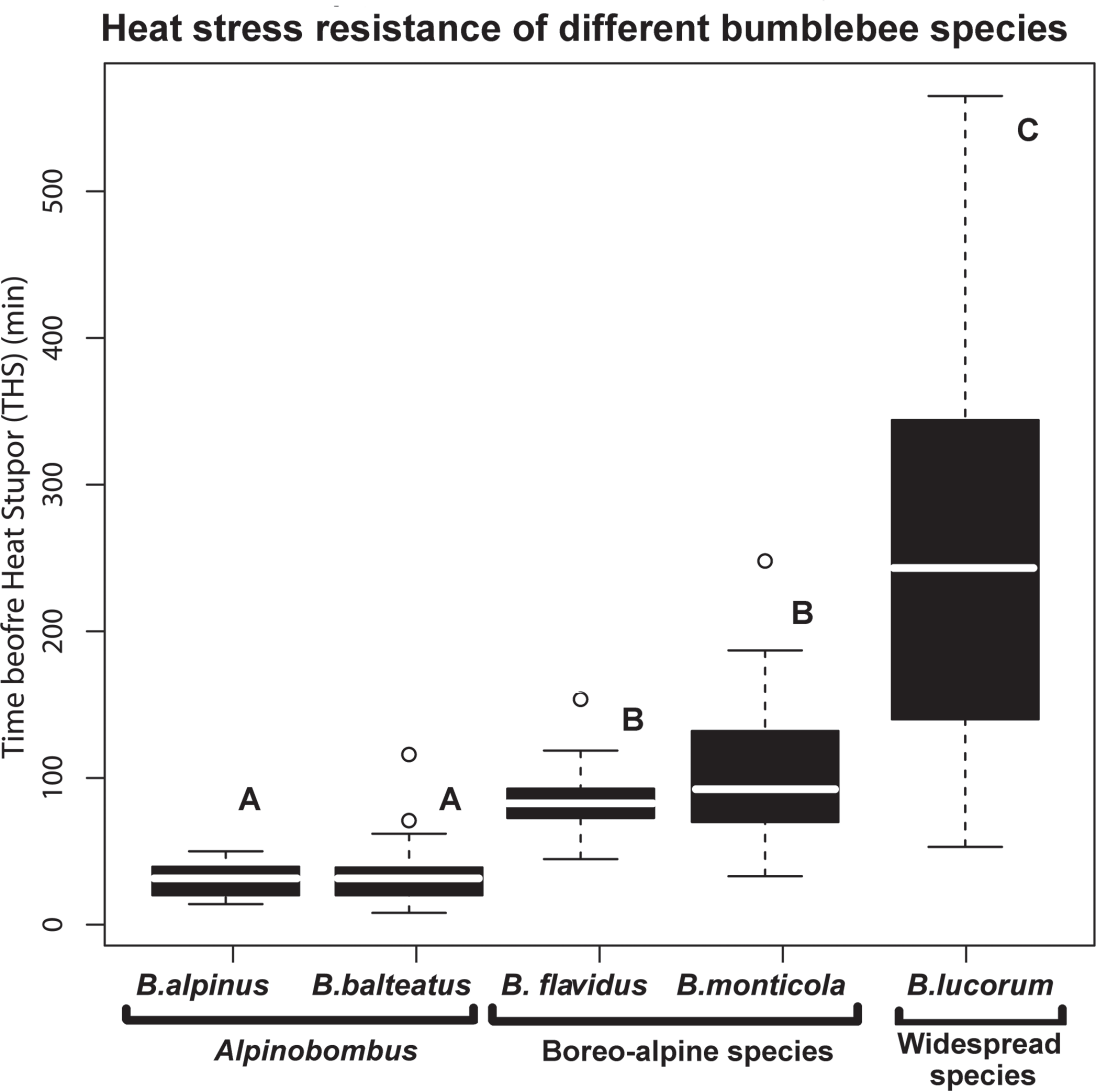
Boxplots of the time before heat stupor. Boxplots of the time before heat stupor (THS) for five bumblebee species: Arctic centred species (A): *Bombus alpinus* and *Bombus balteatus*; Boreo-Alpine species (B): *Bombus flavidus* and *Bombus monticola;* Widespread species (C): *Bombus lucorum*. Circles = extreme values.

Similarly to death ratio results, *B*. *lucorum* has the longest THS (median = 242 minutes) while other species stretch from Boreo-Alpine taxa (intermediate THS: *B*. *monticola* and *B*. *flavidus*) to species with a centred arctic distribution (low THS *B*. *alpinus* and *B*. *balteatus*) (Table 1, Fig. 2).

The Kruskal Wallis test confirms that there are significant differences between species (KW chi^2^ = 92.78, *P* < 0.05). Pairwise statistical analysis (Multiple comparison after Kruskal-Wallis test) show that centred arctic species (*Alpinobombus*) are characterised by a very low similar heat stress resistance (*P* > 0.05, Table 2) while Boreo-Alpine species (*B*. *monticola* and *B*. *flavidus*) have a higher heat resistance (*P* < 0.05, Table 2) than Arctic species but a weaker heat resistance than a widespread and ubiquitous species as *B*. *lucorum* (*P* < 0.05, Table 2).

**Table 2:** Values of the Multiple comparison after Kruskal-Wallis test (Diff means difference) to compare the heat stress resistance (Time before heat stupor) of different bumblebee species (*Bombus*).

**Species**	***B*. *balteatus***	***B*. *lucorum***	***B*. *flavidus***	***B*. *monticola sspp*.**	***B*. *m*. *rondoui***	***B*. *m*. *scandinavicus***
***B*. *alpinus***	Diff = 4.34, P> 0.05	Diff = 97.49, **P<0.05**	Diff = 51.51, **P<0.05**	Diff = 62.85, **P<0.05**	Diff = 57.96, **P<0.05**	Diff = 62.69, **P<0.05**
***B*. *balteatus***	-	Diff = 93.15, **P<0.05**	Diff = 47.17, **P<0.05**	Diff = 58.47, **P<0.05**	Diff = 53.62, **P<0.05**	Diff = 58.35, **P<0.05**
***B*. *lucorum***	-	-	Diff = 45.98, **P<0.05**	Diff = 35.76, **P<0.05**	Diff = 39.53, **P<0.05**	Diff = 34.80, **P<0.05**
***B*. *flavidus***	-	-	-	Diff = 11.20, P>0.05	Diff = 6.45, P>0.05	Diff = 11.18, P>0.05
***B*. *m*. *rondoui***	-	-	-	-	-	Diff = 4.73, P>0.05

Only the P-values < 0.05 were considered significant (Bold).

## Discussion

### Test case

The percentage of mortality (Table 1) of male bumblebees suggests that 40°C resembles the approximate lethal temperature 50% (LT_50_) [16] at least in Arctic centred and Boreo-Alpine bumblebees. However to determine the exact LT_50_, special experiments are required where the animals are exposed to a set of different temperature with equal exposure times and post-treatment observation intervals for all individuals. Further studies are needed to determine this LT_50_ for bumblebee species.

Our results show that the heavyweight Arctic species *B*. *alpinus* and *B*. *balteatus* (Table 1) display a low heat stress resistance (Fig. 2). In contrast, *Bombus monticola*, a lightweight species (Table 1) with a large boreo-alpine distribution, has a higher heat stress resistance (Table 1, Fig. 2). This observation could reflect the Bergmann’s rule [42,43]. According to this rule, species of warm countries are smaller and have a higher ratio surface/volume than species of cold countries. Thus, according to this rule, Arctic species should be large with a low heat stress resistance. However, this rule is valid for endothermic organisms and it is often questioned for ectothermic or optional endothermic organisms. So, we cannot draw conclusions too hasty concerning if the heat stress resistance could be deduced from size. Further physiological and ecological studies are necessary to validate the Bergmann's rule for bumblebees or to highlight other factor as ambient temperature which appears to be a more important factor.

The similar heat stress resistance between *B*. *alpinus* and *B*. *balteatus* could result from their closely phylogenetic relationship [44] or from their identical eco-climatic constraints. *Bombus flavidus*, which is likely the cuckoo species of *B*. *monticola* [45,46], does not have a significantly different heat stress resistance compared to its host. For *B*. *monticola*, our sampling allows taking into consideration two distinct allopatric populations from different eco-climatic regions (*B*. *monticola rondoui* from Pyrenees and *B*. *monticola scandinavicus* from Scandinavia). Our results show that there is no difference in heat stress resistance between these allopatric populations (Table 2). This suggests that the heat stress resistance could be similar between allopatric conspecific populations from different eco-climatic areas. Further pieces of evidence in other species are required to assess the intraspecific variation of heat stress resistance.

Our results strongly suggest that heatwaves could quickly lead to fatal consequences for bumblebee species, e.g. *Alpinobombus* as it has been suggested by Rasmont & Iserbyt [47]. Therefore, the relative importance of heatwaves in the bumblebee decline should be taken into account in future studies on the trigger factor of worldwide bumblebee regression.

### Limits of experiment

Heat resistance could be influenced by many natural factors that can not be controlled in this experimental device [48]: (i) all analysed species have a different phenology and thus it could have been that old males are less resistant than young males. However, according to Terblanche et al. [49], age has no influence on the critical temperature of insects. (ii) The uncontrolled health status of the tested specimen can also have an impact on its heat stress resistance. (iii) The duration of habituation (24 hours at 8°C) may influence the survival time [37,50] but in our comparative method, the experimental conditions are standardised thus the duration of habituation’s influence is negligible. (iv) Experiments were conducted at different times during the day, however, the daily cycles would have no influence on survival time [51]. (v) In this experimental device, flying insects do not have the ability to fly, which is normally the primary cause of increased thoracic temperature with the endothermy for brood incubation [21,29,52]. Thus, the present test conditions could be less stressful than some actual heatwaves. (vi) In the field, insects could adapt a behavior to reduce the effect of overheating such as taking refuge in a fresh place as under a rock or under a flower thereby abandoning their nuptial behavior. These two last parameters are very difficult to achieve in bioassays.

To conclude, this experimental device allows estimating easily the interspecific heat stress resistance of insects in the field or locations close to the field. That could be crucial in the context of the current climate changes. This protocol has the advantage of evaluating the heat resistance of insects almost directly without requiring a step of rearing or a sustained maintenance of specimens. This gives us the opportunity to significantly increase the number of replicates.

## Supporting Information

S1 Dataset Table sampling. Table sampling with all tested specimens and their collection characteristics.
(XLSX)
